# Integrated analyses of the microbiological, immunological and ontological transitions in the calf ileum during early life

**DOI:** 10.1038/s41598-020-77907-0

**Published:** 2020-12-04

**Authors:** Tamsin Lyons, Hanne Jahns, Joseph Brady, Eóin O’Hara, Sinéad M. Waters, David Kenny, Evelyn Doyle, Kieran G. Meade

**Affiliations:** 1 Environmental Microbiology Group, School of Biology and Environmental Science and Earth Institute, University College Dublin, Belfield, Ireland; 2 Pathobiology Section, School of Veterinary Medicine, University College Dublin, Dublin, Ireland; 3 Animal & Bioscience Research Department, Animal and Grassland Research and Innovation Centre, Teagasc, Grange, County Meath Ireland; 4 Department of Agriculture, Food, and Nutritional Sciences, University of Alberta, Edmonton, AB Canada; 5 School of Agriculture and Food Science, University College Dublin, Dublin 4, Ireland

**Keywords:** Mucosal immunology, Animal physiology, Microbiome

## Abstract

Aberdeen Angus calves were sacrificed from immediately post-birth up to 96 days of age (DOA) and ileal samples were collected for microbial, histological and immunological analyses. *Firmicutes* bacteria were established immediately in the ileum of calves after birth and remained the dominant phyla at all time points from birth until 96 DOA. Temporal shifts in phyla reflected significantly increased *Bacteroidetes* at birth followed by temporal increases in *Actinobacteria* abundance over time. At a cellular level, a significant increase in cell density was detected in the ileal villi over time. The innate cell compartment at birth was composed primarily of eosinophils and macrophages with a low proportion of adaptive T lymphocytes; whereas an increase in the relative abundance of T cells (including those in the intra-epithelial layer) was observed over time. The ileal intestinal cells were immunologically competent as assessed by expression levels of genes encoding the inflammasome sensor *NLRP3*, and inflammatory cytokines *IL1A*, *IL1B* and *IL33*—all of which significantly increased from birth. In contrast, a temporal reduction in genes encoding anti-inflammatory cytokine *IL10* was detected from birth. This study provides an integrated baseline of microbiological, histological and immunological data on the immune adaptation of the neonatal ileum to microbial colonisation in calves.

## Introduction

Recent studies focussing on the detection and characterisation of the microbiome that confronts every neonate has led to an expansion in our understanding of the prokaryotic species which live in and on eukaryotes, including humans^1^. In livestock species, characterisation has been more limited but a predominant focus on the gastro-intestinal (GI) tract of ruminants has led to comprehensive characterisation of the initial aerobic and facultative anaerobic bacteria such as *Staphylococcus, Streptococcus, Enterococcus* and *Enterobacteria* which utilise available oxygen and permit the subsequent establishment of anaerobes^2–4^. In the days and weeks following birth the establishment of methanogens, fungi and protozoa occurs^5^. It is now known that the neonatal ruminant GI tract represents a series of unique ecological niches harbouring distinct microbiomes that will play key roles in digestion, health and animal production of the adult animal^6–8^.

In the hours and days following birth, the neonatal calf receives only a liquid diet of colostrum followed by milk which provides early microbes which colonise the intestine^9^. At this stage, a specialised structural adaptation of the ruminal wall—known as the oesophageal groove, routes milk directly to the abomasum and ruminal development does not occur until solid food is introduced. Along with passive transfer of maternal antibodies, colostrum and milk also deliver growth factors (insulin-like growth factor-IGF-1 and hormones) to the neonate which influence gut development by increasing epithelial cell proliferation and villi and crypt growth in the small intestine^10^. Despite the detailed knowledge now available on the prokaryotic complexity within the neonate, how the host recognises, responds and adapts to it over time, remains poorly understood. Furthermore, this initial colonisation has important consequences for multiple complex physiological processes, not least of which is the appropriate development of the immune system, and all of which must be tightly regulated to prevent inflammation-associated tissue pathology and death.

While anatomically formed pre-partum, functional development of the ruminant immune system occurs post-partum. Prior to adaptive immune system development, the neonate principally relies on the innate immune system for protection from infection. It is now recognised that these early host-microbe interactions also contribute to the programming of the immune system^5^. Although the rapid colonisation of the mucosal surfaces including the host intestine by microbial communities might be expected to cause pathological inflammation, the mechanisms preventing uncontrolled inflammation and maintaining homeostasis are not fully understood. Some regulatory strategies have been identified including a mechanism by which microorganisms produce signals to promote maturation of regulatory immune cells and prevent hyper-activation of the immune response^11,12^. The term ‘inflammatory anergy’ is used to describe the temporary silencing of inflammatory responses during the establishment of a protective microbiome. However, the empirical evidence for these mechanisms are lacking in non-model organisms.

In cattle, the richest site of immune cell deposition along the intestine is in the ileum. The ileum is part of the small intestine which in its submucosa contains a high concentration of lymphoid nodules called Peyer's patches. These nodules form the gut-associated lymphoid tissue that is referred to as the “immune sensor” of the intestine^13^. These immune centres lined by follicle-associated epithelium (FAE) are critical for the distinction between pathogenic and commensal bacteria. Part of the epithelial layer contains specialised M cells which function as transporter cells (luminal proteins as well as antigens) from the intestinal lumen to the immune cells, and thereby serve as a gateway to tolerance or immune activation. These interactions are mediated via cellular receptors on resident epithelial cells as well as immune cells, referred to as Toll-like receptors (TLRs). Epithelial cells lining the GIT of germ-free animals had reduced expression of TLRs, suggesting commensal gut microbes promote the expression of these receptors^14^. Lotz et al. reported that down-regulation of the TLR4 signalling pathway which detects bacterial LPS prevents an inflammatory response^15^. One of the few studies conducted in calves describes down-regulated mRNA expression levels for several TLRs and antimicrobial peptides in the small intestine in the weeks after birth compared to calves at 6 months^16^. Additionally, *IL1* and *IL6* gene expression in the calf jejunum has been recently shown to be affected by method of birth^17^. In cattle, FAE of Peyer's patches have a critical role in trafficking not only colostral leukocytes into neonatal circulation^18^ but also infectious disease antigens including mycobacteria and thereby play a critical role in the immune response to these agents^19^. A number of problematic enteric infections in cattle are known to target the M cells in the intestine^20–22^, therefore suggesting that host–pathogen interactions in this region are of critical importance. However, there is limited knowledge on the pattern of tissue and immune system maturation in early life and how colonising microorganisms interact with the host to promote homeostasis.

The aim of this study was to comprehensively characterise the combined changes in microbial diversity and the development of the immune response occurring in conjunction with morphological development of the ileum in calves, from birth until 96 days of age. Understanding the integration, orchestration and ontogeny of host-microbe interactions in early life is key to the design of new strategies to protect calves against opportunistic bacterial and viral infections.

## Results

### Effect of age on ileal bacterial community dynamics of new-born calves

Bacterial 16S amplicon sequencing generated 29,169,392 total sequences and after initial processing, an average of 788,362 high-quality sequences were obtained per ileal sample, with read numbers ranging from 207,609 to 3,071,093 (median = 583,507). The vast majority of reads were successfully classified with only 0.39% 3.4% at 20.87% remaining unclassified at phylum, family and genus levels, respectively.

**Figure 1:**
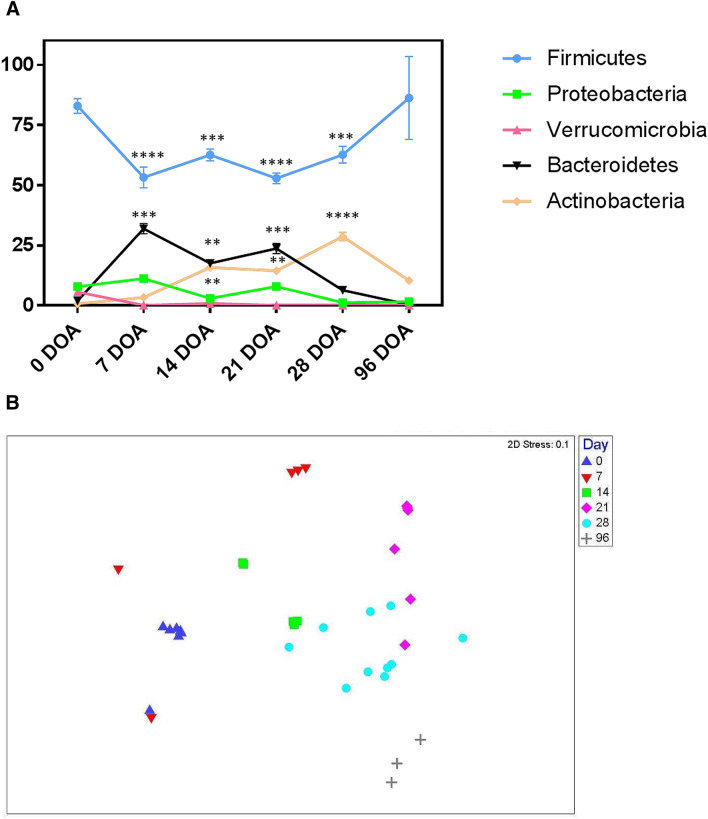
(**A**) Mean relative abundance (%) of sequences assigned to dominant bacterial phyla present in the ileum of calves at 6 sampling time points (birth—0, 7, 14, 21, 28 and 96 days of age, DOA). N = 3, mean ± SEM. Statistical significance is denoted as *, **, *** and **** for P values < 0.05, < 0.01, < 0.001 and < 0.0001, respectively. For clarity of interpretation, only significant changes from birth (0 DOA) are shown. (**B**) Non-metric multidimensional scaling (nMDS) plot showing clustering of bacterial community structure, at genus level, in the ileum of calves based on age.

Twenty bacterial phyla were identified in the ileum of calves throughout early life (Supplementary Table 1). However, only 5 of those were present at abundances > 5% at any one sampling time point and are shown in Fig. 1A. *Firmicutes* dominated the ileum of new-born calves and these remained the most dominant phyla across the 96 day sampling window, accounting for 83% of total bacterial sequences detected at birth and ranging from 53–86% across time points up to 96 DOA. *Proteobacteria* (7.86%)*, Verrucomicrobia* (5.61%) *Bacteroidetes* (1.96%), *Actinobacteria* (0.81%), *Chlamydiae* (0.39%) and *Synergistes* (0.28%) phyla combined to make up the remaining fractions of bacterial sequences (combined total 17%) at birth. At family level, *Streptococcaceae* were strongly dominant with 76.21% of all bacterial sequences assigned to this family. Other families present at high relative abundance included *Verrucomicrobiaceae* (5.6%), *Pasteurellaceae* (3.37% ± 0.33%), *Enterobacteriaceae* (2.12%), *Campylobacteriaceae* (1.59%), *Lachnospiraceae* (1.28%), *Veillonellaceae* (0.53%) and *Synergistaceae* (0.28%). Together these 8 families comprised approximately 91% of all bacterial sequences detected. At birth, the majority of all sequences (71.21%) were assigned to the genus *Streptococcus,* with *Akkermansia* (5.6%)*, Lactococcus* (4.92%)*, Gallibacterium* (2.9%)*, Escherichia* (2.11%) and *Campylobacter* (1.59%) the next most relatively abundant genera. All other genera were present in lower relative abundances, each comprising less than 1% of total bacterial sequences obtained from the ileum samples.

Non-dimensional multiscaling (nMDS) and PERMANOVA were used to determine if significant differences existed between bacterial community structures in the ileum of calves across different age groups up to 96 DOA. Age-related clustering of bacterial community profiles was observed at phylum, family and genus level, but only clustering at genus level is shown in the nMDS plot displayed in Fig. 1B. Bacterial community structures in calves at 96 DOA can be observed to cluster quite a distance away from communities at all other time points. Similarly, bacterial communities in new-born calves (0 DOA) and calves at 7 DOA were very distinct from all other time points, with the exception of a couple of outliers. PERMANOVA analysis confirmed that bacterial community structures present in the ileum of calves differed at all 6 time points as observed on the nMDS plot (*P* = 0.0001).

Bacterial phyla contributing to differences in community structures at the different time points were identified by similarity percentage (SIMPER) analysis (Supplementary Table 2). Although *Firmicutes* was the dominant phylum at all times sampled, changes in the relative abundance of this phylum were not the main driver of dissimilarity in community structures. The main contributor to differences in community structures at most time points was another dominant phylum, *Bacteriodetes.* The relative abundance of this phylum increased from birth to 7 DOA and this explained 19.7% of the dissimilarity between community structures of new-born and calves at 7 DOA. Decreases in the relative abundance of *Verrucomicrobia, Chlamydiae, Synergistetes* and *Fibrobacteres* also contributed to the dissimilarity in bacterial community structures at the different time points. The relative abundance of *Proteobacteria* declined from birth until 28 DOA and while this phylum didn’t contribute to any differences observed at birth, it did contribute after that point. In contrast, the relative abundance of *Actinobacteria* increased from birth to 28 DOA but had declined by 96 DOA when it explained ~ 11% of the dissimilarity observed between bacterial communities in 28-day and 96-day old calves.

At genus level, relative abundance of *Streptococcus, Gallibacterium, Akkermansia* and *Lactococcus* in the ileum was lower at 7 DOA compared to birth, while relative abundances of *Bacteroides, Prevotella, Subdoligranulum* and *Faecalbacterium* were all increased. These bacteria explained roughly 15% of the dissimilarity between bacterial community structure in the ileum of new-born calves and week-old calves. Sequences assigned to the genera *Subdoligranulum* and *Bacteroides* had decreased by 14 DOA relative to other bacteria. *Bifidobacterium, Sharpea, Olsenella, Veillonella, Prevotella* and *Gallibacterium* all increased in relative abundance at 14 DOA and together explained over 15% of the dissimilarity between bacterial community profiles at 7 DOA and at 14 DOA.

At 21 DOA, the relative abundance of *Streptococcus, Lactococcus, Veillonella* and *Clostridium *sensu stricto had all decreased whereas *Succinivibrio, Allobaculum, Faecalibacterium, Sutterella* and unclassified genera from *Alphaproteobacteria* and *Bacteroides* had all increased. When combined, these genera accounted for 15% of the dissimilarity in ileum bacterial structure between time points. At 28 DOA, the relative abundance of *Succinivibrio, Allobaculum, Faecalibacterium, Prevotella* and unclassified *Alphaproteobacteria* and *Bacteroides* again decreased while *Romboutsia* and *Sharpea* increased*.* Finally at 96 DOA, there were decreases in the relative abundances of *Bifidobacterium, Sharpea* and *Streptococcus* while relative abundances of *Saccharofermentans, Lysinibacillus, Turicibacter, Escherichia* and unclassified genera from *Ruminococcaceae, Lachnospiraceae* and *Candidatus Saccharibacteria* all increased and accounted for almost 14% of the dissimilarity calculated in bacterial community structure in the ileum of calves 28 days after birth (pre-weaning) and the ileum of calves 96 days after birth (post-weaning).

At phylum level, bacterial richness and diversity (based on the Shannon–Wiener index) decreased between birth and 7 DOA (*P* = 0.0005 and *P* = 0.003 respectively). Bacterial richness returned to levels similar to those in new born calves on 14 DOA (*P* = 0.004) (Supplementary Table 3). At the genus level, bacterial richness, evenness and diversity also dropped between birth and 7 DOA (*P* = 0.0005) and fluctuated thereafter. Interestingly, despite these fluctuations no significant differences (*P* > 0.05) were observed in bacterial richness, evenness or diversity at phylum or genus level between the ileum of new-born calves and 96-DOA calves.

### Morphological changes in the ileum of new-born calves from birth to 96 DOA

**Figure 2:**
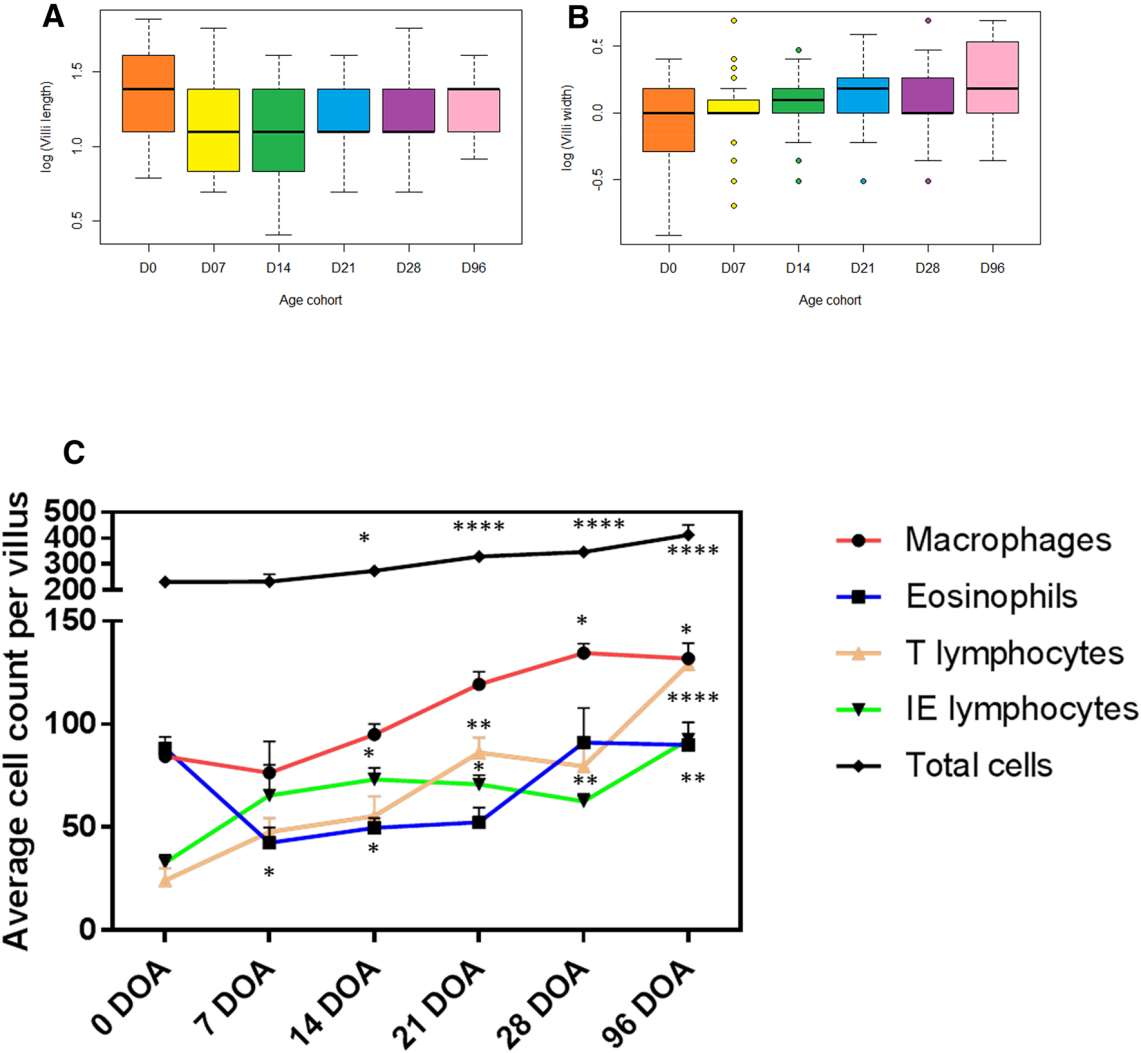
(**A**) Villi length and (**B**) width (mm) in the ileum of calves at 6 sampling time points (birth—0, 7, 14, 21, 28 and 96 days of age, DOA). (**C**) Average immune cell counts (macrophages, eosinophils, T lymphocytes), including the epithelial layer (intra-epithelial lymphocytes, IEL) per villus. N = 3 calves per group. Statistical significance is denoted as *, **, *** and **** for P values < 0.05, < 0.01, < 0.001 and < 0.0001, respectively. For clarity of interpretation, only significant changes from birth (0 DOA) are shown.

The length of villi in the ileum of calves significantly decreased between birth and 7 DOA (*P* = 0.008, Fig. 2A) but there was no significant difference in villus length at 96 DOA compared to new-born calves (*P* = 0.8, Fig. 2B). The width of the villi was similar across all age groups.

Overall, the average number of immune cells per ileal villi markedly increased from 14 DOA with significant increases at 14 (*P* < 0.05), 21 and 28 DOA (*P* < 0.0001) (Supplementary Table 4). By 96 DOA, total cell numbers almost doubled in number showing significant sustained cellular infiltration into the villi (*P* < 0.0001, Fig. 2C). Neutrophils were observed in low numbers in the lamina propria mucosa with occasional exocytosis in two animals each at D7, D14 and D96 and one animal each at D21 and D28 (data not shown). Eosinophils and macrophages were found distributed evenly throughout the lamina propria mucosa at the level of the villi and crypts (Fig. 2C), and these innate cells were the most abundant. In addition, single macrophages were observed in moderate numbers in the submucosa overlying the Peyer’s patches and in the follicles in all age groups. A significant increase in macrophage number was detected at 28 and 96 DOA compared to birth (*P* < 0.05) and macrophages remained the most abundant cell type throughout the 96 day sampling period. In contrast, eosinophils displayed s significant decrease at 7 and 14 DOA relative to birth (*P* < 0.05, Fig. 2C).

Numbers of adaptive cells were lowest at birth but T lymphocyte populations steadily increased from birth with significant increases at 21, 28 and 96 DOA in T cells (*P* < 0.01). Adaptive T-cells showed a similar mucosal distribution and also infiltrated the mucosal epithelial lining cells (referred to as intra-epithelial lymphocytes). From 14 DOA, the number of intra-epithelial lymphocytes significantly increased up to and including 96 DOA (*P* < 0.01, Fig. 2C). Moderate numbers of T-cells were seen in the submucosa overlying the Peyer’s patches and few single T-cells were seen within the follicles at D28 and D96. In the lamina propria mucosa only single B-cells were detected in all age groups and these cells were therefore not included in the cell count. Dilated lymphatics in the villi were occasionally filled with B-cells at D7, D14 and D28. Further, B-cells were diffusely seen in the lymphoid follicles of the Peyer’s patches. In addition, moderate numbers of B-cells were also observed in the submucosa overlying the Peyer’s patches (data not shown). In the dome area single T-cells were observed at day 0 which increased with age (Supplementary Fig. 1). Moderate numbers of B-cells populated the dome area and infiltrated the FAE. These numbers increased slightly with age as did the number of B-cells infiltrating the FAE. Similarly, macrophages within the lymphoid tissue of the dome increased in number with age.

**Figure 3:**
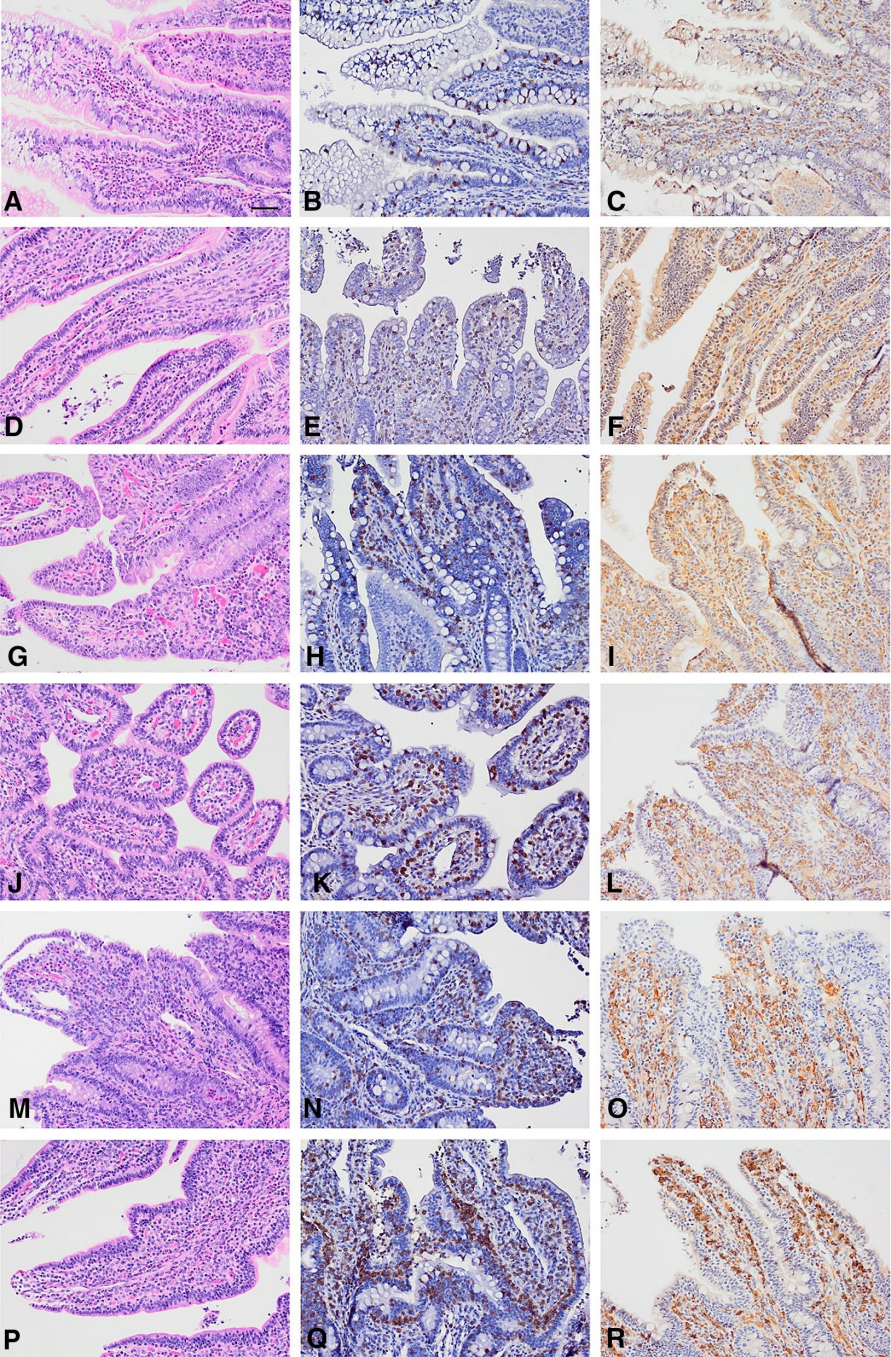
*Calf ileum plate*; Representative images of the ileal villi of calves from 6 different age groups; birth—0 (a to c), 7 (d to f), 14 (g to i), 21 (j to l), 28 (m to o) and 96 (p to q) days of age (DOA). First column H&E staining. Second column, Anti-CD3 immunohistochemical staining (brown), counterstained with Mayer’s haematoxylin. Third column, Anti-Iba1 immunohistochemical staining (brown), counterstained with Mayer’s haematoxylin. Scale bar = 50 µm.

Representative images of the ileal villi of calves are shown in Fig. 3 across age groups from birth—0 (a to c), 7 (d to f), 14 (g to i), 21 (j to l), 28 (m to o) and 96 (p to q) DOA. The number of eosinophils infiltrating the lamina propria mucosa of the villi is decreased by approximately 50% on 7 and 14 DOA compared to at birth. The number of eosinophils increases on 28 DOA with highest levels seen at 96 DOA. T-cells, observed in the lamina propria mucosa and in the mucosal epithelium, clearly increase with age. The numbers of macrophages infiltrating the lamina propria mucosa of the villi also increase in number until 28 DOA of age.

### Correlations between immune cell abundance and relative abundance of bacteria in the ileum of calves during early life

Correlations between the three dominant immune cell types (eosinophils, macrophages and T lymphocytes) and dominant bacteria revealed some informative correlations at both phylum and genus level. Average T lymphocyte number in the ileal villi was positively correlated with the relative abundance of phyla *Candidatus Saccharibacteria* but negatively correlated with *Verrucomicrobia*. In contrast, average eosinophil number per villi was negatively correlated with the relative abundance of the phyla *Bacteroidetes* (Table 1).

**Table 1:** Correlations between the relative abundance of sequences assigned to bacterial phyla in the ileum and the percentage of immune cells (macrophages, T cells & eosinophils) present in the ileal villi of calves over the first 96 days of life.

Phylum	Eosinophil	Macrophage	T cell
*Proteobacteria*	− 0.061	− 0.342	− **0.414**
*Actinobacteria*	− 0.025	0.229	0.130
*Bacteria_*unclassified	0.134	**0.545**	**0.544**
*Bacteroidetes*	− **0.596**	− 0.246	− 0.201
*Candidatus_Saccharibacteria*	0.199	0.318	**0.645**
*Chlamydiae*	0.195	0.068	0.204
*Chloroflexi*	**0.507**	0.395	0.346
*Elusimicrobia*	− 0.117	− 0.139	− 0.035
*Fibrobacteres*	0.252	0.181	0.010
*Firmicutes*	− 0.037	− 0.048	0.009
*Fusobacteria*	− 0.113	− **0.421**	− **0.530**
*Lentisphaerae*	− 0.228	− 0.202	− 0.218
*Planctomycetes*	0.302	0.292	**0.424**
*Proteobacteria*	− 0.269	− 0.292	− 0.378
*SR1*	0.126	− 0.214	− 0.237
*Spirochaetes*	0.309	0.304	0.158
*Synergistetes*	− 0.080	− 0.352	− **0.462**
*Tenericutes*	0.294	− 0.226	− **0.472**
*Verrucomicrobia*	0.118	− **0.482**	− 0.661

Pearson’s correlation coefficients (r) are given with r < 0 indicating a negative correlation and r > 1 indicating a positive correlation. Strong correlations with r > 0.4 are shown in bold.

The abundance of intraepithelial T cells, which play an important role in pathogen resistance and tissue tolerance, was negatively correlated with the relative abundance of the phyla *Proteobacteria*, *Verrucomicrobia, Tenericutes, Synergistes* and *Fusobacteria* in the ileal digesta (Supplementary Fig. 2). A low positive correlation was detected between intraepithelial T cell abundance and the relative abundance of unclassified bacteria, but no other statistically significant correlations were observed.

At genus level, average T cell number in the villi was positively correlated with the relative abundance of *Olsenella, Saccharofermentans, Turicibacter* and unclassified genera of *Candidatus Saccharibacteria* and *Lachnispiraceae* and a negative correlation with *Lactococcus, Streptococcus, Succiniclasticum, Campylobacter, Gallibacterium* and *Akkermansia* (Supplementary Fig. 3)*.*

T cell abundances in the intraepithelial layers were also negatively correlated with many of the same bacterial genera (*Lactococcus, Streptococcus, Succiniclasticum, Campylobacter, Gallibacterium* and *Akkermansia*). Some moderate positive correlations were observed between T cell populations in the intra-epithelial layers of the villi and the relative abundance of *Turicibacter* and unclassified genera of *Lachnospiraceae* and *Candidatus Saccharibacteria* (Supplementary Fig. 4). In contrast, eosinophil abundance in the villi had no strong positive correlation with any bacteria genera and showed only moderate negative correlations with *Blautia* and *Subdoligranulum.*

Likewise macrophage abundance in the villi showed no strong positive correlations with the abundance of specific bacterial genera in the ileum digesta, and only moderate negative correlations were observed with *Subdoligranulum* (Supplementary Fig. 3). No statistically significant correlations were detected between the remaining bacterial phyla or genera and the abundance of any of the immune cells counted in the villi of calves between birth and 96 DOA.

### Host gene expression in ileum over time

**Figure 4:**
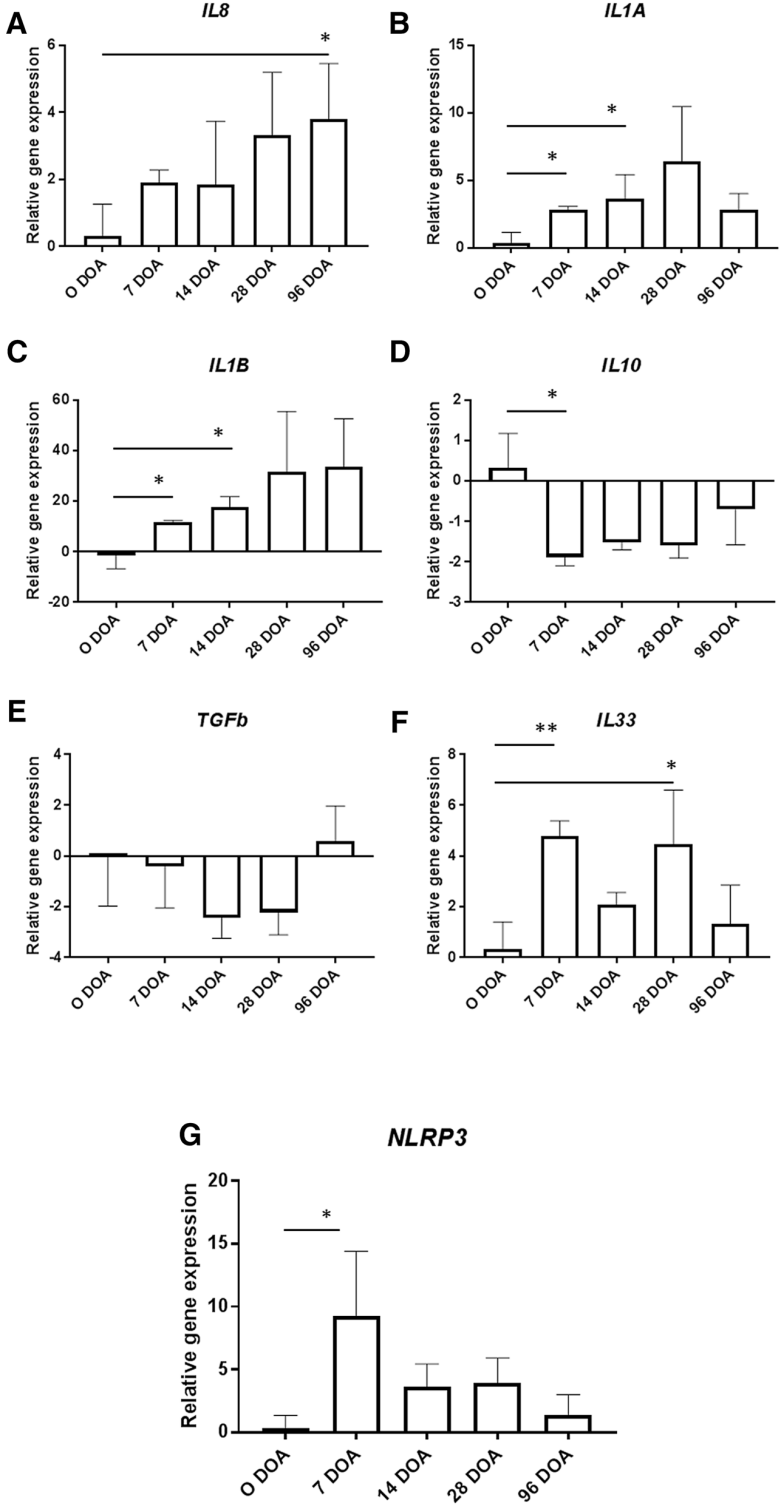
Relative expression of immune genes (**A**) *IL8*; (**B**) *IL1A*; (**C**) *IL1B*; (**D**) *IL10*; (**E**) *TGFB*; (**F**) *IL33* and (**G**) *NLRP3* in the ileum of calves at birth (D0), day 7 (D07), day 14 (D14), day 28 (D28) and day 96 (D96). N = 3 calves per group. Statistical significance is denoted as *, **, *** and **** for P values < 0.05, < 0.01, < 0.001 and < 0.0001, respectively. For clarity of interpretation, only significant changes from birth (0 DOA) are shown.

Expression levels of genes encoding cytokines associated with pro- and anti-inflammatory pathways of the host immune system were measured by qPCR. A consistent trend of increased expression of the chemokine *IL8* was observed from birth and the increase reached statistical significance at 96 DOA (Fig. 4). A similar increase in expression from birth was also detected for the members of the IL1 family of proinflammatory cytokines. Expression of *IL1A* was significantly increased at 7 and 14 DOA, relative to birth; and *IL1B* expression was significantly increased at the same time points (*P* < 0.05). In contrast to this pattern of activation, both anti-inflammatory genes analysed showed a distinct reduction in levels present from birth with a significant reduction in *IL10* by 7 DOA. Interestingly, a biphasic response pattern was detected for the gene encoding cytokine *IL33* with two significant increases from birth to 7 and 28 DOA, respectively (*P* < 0.05). Expression of the inflammasome sensor *NLRP3* gene was also significantly increased at 7 DOA relative to birth (*P* < 0.05). Pearson’s correlation analysis showed no strong relationships between the abundance of different immune cells and the expression of genes encoding cytokines in the ileal villi (Supplementary Fig. 5A). However, a strong positive correlation was detected between intraepithelial T cell abundance and the expression of *IL1β* and a negative correlation between IEL T cell abundance and *IL10* (Supplementary Fig. 5B).

## Discussion

Research on the ruminant gut microbiome has increased in recent years, with a wealth of information now available in relation to microbial abundance and environment-related shifts in bacterial populations^5,16,23–25^. However, establishment of the site-specific microbiome in conjunction with structural post-natal adaptations, as well as the implications for neonatal immunity have to-date generally been studied as separate aspects of ruminant biology. The majority of studies investigating the developmental ontogeny of the digestive tract in the neonatal calf have tended to focus only on a single time point and usually on the rumen, which is understandable given that it accounts for approximately 70% of the entire stomach of the adult animal and represents a unique digestive environment. Furthermore, the reliance on analysis from faecal samples as a proxy to indicate which microbes may be present in the lower GI tract^26–28^ does not reveal regional specification and it limits our ability to understand region-specific relationships.

### Initial colonisation

The initial microbial colonisation of the GIT in the neonatal calf is unique, as at this stage the rumen is non-functional and in this regard, the vast majority of what is known has been extrapolated from mouse and other monogastric animal models. Although the basic tissue structure of the GI tract is formed during gestation, rapid morphological and functional development occurs immediately after birth^29,30^. From a purportedly sterile environment in utero, the peri-natal calf must immediately adapt to an extra-uterine environment of immense microbial complexity. In a range of animals, *Firmicutes, Proteobacteria* and *Bacteroidetes* are known to inhabit the vaginal canal of the dam pre-parturition and account for the majority of bacteria present in the neonate intestine immediately after birth^31,32^, consistent with the findings for the ileal digesta in this study. In this study, *Firmicutes* was present in the highest relative abundance in new-born calves and this agrees with the bacterial profile in the ileum of young calves reported in other studies^23^ and also in goats^33^. Furthermore, the majority of other microorganisms detected in the ileum immediately after birth in the current study belonged to the phyla *Proteobacteria*, *Verrucomicrobia*, *Bacteroidetes*, *Actinobacteria*, *Chlamydiae* and *Synergistes*. A similar composition was reported in the ileum of mature cows^8^. This vertical transmission suggests that there is a strong maternal influence on microbial composition in the gut in early life. Yeoman et al. compared the microbiota in the lumen of calves to the microbiota in maternal colostrum, udder skin, and vaginal scrapings and found 46% of microorganisms detected in the calves were also detected in one of the maternal sources. In this study, 8 families (*Streptococcaceae, Verrucomicrobiaceae, Pasteurellaceae, Enterobacteriaceae, Campylobacteriaceae, Lachnospiraceae, Veillonellaceae* and *Synergistaceae*) together comprised approximately 91% of all bacterial sequences detected in the ileum of young calves, with *Streptococcaceae* by far the most dominant^9^. At the genus level, members of the genus *Streptococcus* were found to be dominant. It has been suggested that initial colonisers such as *Streptococcus* and *Enterococcus* utilise available oxygen in the gut creating an anaerobic environment which facilitates ileal colonisation by strict anaerobic bacteria^5^. At genus level, the majority of all sequences are assigned to 6 genera all of which are anaerobes, facultative anaerobes or microaerophilic microorganisms and associated with the gut environment. The most abundant, *Streptococcus* (71.21% ± 4.47%) are known to reside in the gut of young calves and be dominant in the 24 h after birth^34^.

In humans, *Bacteroides* and *Bifidobacterium* are two of the main gut bacterial genera present and contribute to development of immunotolerance to commensal organisms^35^, which demonstrates the importance of host-microbial interactions during early life to the developing gut and immune system. However, our data indicated that *Bacteroides* and *Bifidobacterium* represented less than 1% of the total bacterial community, indicating distinct species-specific differences in colonisation. In calves, *Firmicutes* remained the most abundant phylum at all time points within ileal digesta and contributed only modestly to the overall dissimilarity in bacterial community structure between calves from different age cohorts. Although low in abundance, *Bacteroidetes* were the main contributors to dissimilarity between the bacterial community structure in new-born calves and week-old calves, increasing in relative abundance between these time points. *Bacteroidetes* produce glycoside hydrolases for glycan degradation and therefore this increase after birth may be linked to increased digestive requirements in the host^36^. This indicates that host-microbe interactions and the mechanisms prompting GI tract development and stimulating immunotolerance may be species-specific and therefore it cannot be assumed that findings from human or rodent studies apply to interactions taking place in the ruminant gut.

### Immunological development

Immune cells within the Peyer’s patches, as well as the associated epithelium are well endowed with pattern recognition receptors including Toll-like receptors which lead to cytokine expression and the production of host defence peptides^37^, and it is these functional molecules that determine the course of the immune response. However, regulation of innate immune responses in the neonate has been suggested as one mechanism by which the host avoids unnecessary inflammatory responses during the early stages of colonisation commensal microbiota. In addition to innate mechanisms, the development of anti-inflammatory Th2 type responses, anergy and T cell deletion have all been described as contributing to immunological tolerance^38^. This immune tolerance is important to allow colonisation with beneficial non-pathogenic bacteria^39,40^, establishment of homeostasis and also to protect the mucosal tissue from detrimental inflammatory responses.

NLRP3 is a member of multi-protein innate immune complex known as the inflammasome which senses a variety of endogenous and environmental stimuli, and thereby regulates homeostasis or damage control^41^. The enhanced expression of *NLRP3* suggests that it plays a conserved regulatory role in ileal function. Innate cells like macrophages are also key regulators of intestinal homeostasis^42^ and they were detected here in large numbers in the ileum of calves immediately after birth. The increased number of macrophages shown here infiltrating the lamina propria mucosa of the villi are a likely source of the cytokines which are significantly increased. *IL1A* and *IL1B* encode key inflammatory proteins which were increased in the ileum in this study, and their expression is known to be mediated via the NLRP3 inflammasome. The potent chemotactic *IL8* gene is increased and reaches statistical significance at 96 DOA. TGFβ is a strong inducer of tolerogenic responses and the expression of the anti-inflammatory cytokine *IL10* was significantly reduced by 7 DOA showing a role at birth. This suggests that IL10 could play a role in modulating inflammation in the neonatal ileum. A significant influx of eosinophils to the ileum was recorded over time, with maximum numbers identified at birth. In the villi, the section of the ileum with closest contact to the external environment and microbial communities, eosinophils accounted for roughly one third of total host cells present. Although the presence of eosinophils in the neonatal ileal villi has not been previously reported in any species, regulatory subsets of these cells have been recently reported. Eosinophils have now been shown to be important for the regulation of the gut microbiota as well as the development of regulatory T cells^43,44^. A new appreciation of the breadth of eosinophil function is now starting to emerge, including their function in the intestine^45^ as key regulators of homeostasis^46^. IL-33 is a key mediator of eosinophil recruitment as well as regulatory T cell expansion^47^, thereby promoting Th2-biased immune responses. Therefore the significantly enhanced expression of this cytokine could play a role in this regard in cattle.

This influx of innate cells and the obvious presence of immune cells in the ileum supports previous reports that the ileum is one of the key sites of immune-microbe interaction in the mammalian gut^13,48–50^. From an adaptive cell viewpoint, T cells were present in low relative abundances in the ileum villi of new-born calves compared to innate cells but T cell numbers increased markedly at 7, 14 and 21 DOA. In our study the eosinophil and T cell population show an inverse relationship and this may be regulated by the eosinophil population^51^.

### Host–microbe interactions

Members of the *Bacteroidetes* genus have been shown to have a role in inducing regulatory T cells which promote epithelial repair, promote tolerance to microbes and initiate suppression of immune responses to self and bacterial antigens^52^. In agreement with these findings, in the current study it was recorded that relative abundance of *Bacteroidetes* in the ileum was positively correlated with the abundance of T cells. T cells in the intraepithelial spaces of the intestine provide a first line of defence against invading bodies at the barrier and are linked to pathogen resistance and tissue tolerance through cytotoxic T cell-related and anti-inflammatory molecules^53,54^. Dysregulation of intraepithelial lymphocytes is correlated with loss of mucosal barrier integrity, susceptibility to infections and chronic inflammatory processes. In this study, intraepithelial T cell populations in the ileum markedly increased at 7, and again at 28 DOA. Fluctuations could represent attempts to balance cytotoxic responses triggered by the influx of microorganisms with tissue tolerant responses necessary to avoid prolonged inflammation of the tissue and subsequent tissue damage.

## Conclusion

Distinctive species-specific features of ileal tissue, and specifically in Peyer’s patches have been described in terms of cellular composition between mouse and humans^13^. Here age-related structural, cellular and molecular changes associated with initial colonization and adaptation of the neonatal bovine ileum have been documented for the first time. Neonatal ruminants are highly susceptible to a variety of viral and bacterial enteric infections within the first few weeks of life^55^ and the role of the gut microbiome in priming the morphological and immune development of the host GIT is crucial^56^. Here we document the presence of large numbers of innate immune cells (eosinophils ad macrophages) in the ileum as an important portal of host-microbe interaction and education of the neonatal immune system. Maladapted early neonatal immune system development could have negative consequences for lifetime immunity^57,58^ and therefore these integrated findings provide an important baseline for future intervention studies. Appropriate activation of the NLRP3 inflammasome and the expansion of eosinophils in ileal tissue are likely to be important in terms of regulating maladaptive inflammation immediately after birth and warrants further investigation.

## Methods

### Experimental design

All procedures described were approved by the Teagasc Animal Ethics Committee and conducted under experimental license from the Irish Health Products Regulatory Authority in accordance with the Cruelty to Animals Act 1876 and the European Communities (Amendments of the Cruelty to Animals Act 1876) Regulations, 1994. This research was carried out as part of a larger study designed to investigate the establishment and development of the rumen microbiome in early life. Full details of this animal model has been recently published^59^. Briefly, commercially purchased 18-month-old oestrous synchronised Aberdeen Angus heifers (n = 21) were artificially inseminated with frozen thawed semen from one Aberdeen Angus sire to provide 70 calves. Calves were assigned at random to one of six age groups. For this study calves were sampled following euthansia at 0 (n = 11), 7 (n = 8), 14 (n = 9), 21 (n = 9), 28 (n = 9) and 96 days of age (n = 9) DOA). Calves were born over four replicated periods; December 2014 (n = 13), January 2015 (n = 27), May 2015 (n = 14), July 2015 (n = 11), and treatments were assigned as evenly as possible across all replicates. Pregnant heifers were held together in a pen until immediately prior to calving and induced to calve by administration of 2 ml prostaglandin F2_α_ analogue (Estrumate, Merck), to facilitate a staggered calving schedule. Calves not assigned to a D0 treatment were allowed to suckle their dam for 48 h post birth at which point they were removed and penned individually. Calves were then artificially reared on milk replacer (5L/d), offered as two equal feeds and offered concentrates from 7 DOA. Calves to be euthanised at 96 DOA were weaned at 8 weeks, when they were consuming 1 kg concentrates per day for 3 consecutive days. Subsequently they were offered 2 kg concentrate per day, with ad libitum access to water and hay.

Following euthanasia, the gastrointestinal tract was removed, and samples of ileal content were collected using sterilized and RNase-free instruments, and immediately snap-frozen in liquid nitrogen. Cross sections of the ileum were washed in PBS and snap frozen in liquid nitrogen. These samples were subsequently stored at − 80 °C. A cross section from the ileum was fixed in 10% neutral buffered formalin. The tissues and content samples were collected within 20 min of euthanisation.

### DNA extraction and microbial community analysis

The room was cleaned before sampling commenced and the area was cleaned again in between each calf. Each calf was sampled in isolation using sterile scalpel blades. All surfaces and sampling equipment were treated with boiling water, ethanol and RNase inhibitor prior to the processing of samples from each calf. Sterile vessels were used to store all samples. Day 0 calves were taken for sampling immediately after birth without further contact with the cow or the external environment.

DNA was extracted from ileal content samples collected from 37 animals at 6 sampling time points; 0 (n = 8), 7 (n = 5), 14 (n = 5), 21 (n = 6), 28 (n = 10) and 96 (n = 3) DOA, using the PCSA method with phenol chloroform added before the bead beating step ^60^. The DNA concentration and purity were assessed using a Nanodrop spectrophotometer (Thermo Scientific, Waltham, MA) and all samples had a 260/280 value of > 1.8 and a 260/230 value > 2. Purified DNA was stored at − 20 °C.

Bacterial community structures were determined using amplicon sequencing on an Illumina Miseq platform (Illumina, San Diego, CA), using the method described by Kozich et al. (2013). Briefly, 1 μL of purified DNA was added to a well on a 96-well PCR plate already containing 17 μL of Accuprime Pfx Supermix (Invitrogen, Thermo Fisher Scientific, Dublin, Ireland) and 2 μL of a primer set targeting the V4 region of the 16S rRNA gene^61^. The PCR conditions consisted of a hot start at 95 °C for 2 min, followed by denaturation at 95 °C for 20 s, annealing at 55 °C for 15 s, and extension at 72 °C for 5 min (30 cycles) with a final extension at 72 °C for 10 min. The PCR products were visualized on a 1.2% (w/v) agarose gel (Roche Diagnostic, Basel, Switzerland); PCR products were then purified, and concentrations normalized using the SequalPrep Normalization Plate Kit (Invitrogen) according to manufacturer’s instructions and an amplicon pool was created using 5 μL of PCR product from each sample. The concentration of the pool was then determined using a Qubit fluorometer and dsDNA kit (Thermo Fisher Scientific) to ensure that at least 100 ng of DNA was present. The amplicon pool was then sent to the Centre for Genomic Research, University of Liverpool (Liverpool, UK), for sequencing on an Illumina Miseq platform. Negative controls were included in library preparation for amplicon sequencing. After PCR the negative controls, like all other samples, were evaluated by gel electrophoresis and were shown to contain no DNA indicating that reagents and materials used up until this point were not introducing contaminants.

MiSeq sequencing data were initially processed using the mothur program v.1.32.1^62^. Illumina adapter sequences were trimmed by cutadapt ver.2.1.1 using option -O 3^63^. Sickle ver.1.2^64^ was used to further trim the data with a quality score of ≥ 20. Reads < 10 bp after trimming were removed. Each read was then trimmed to a maximum of 275 bp and ambiguous bases were removed. Sequences which contained homo-polymer runs > 8 bases were discarded. After trimming, identical sequences were grouped into unique sequences. Chimeric sequences were identified using the UCHIME algorithm^65^ within mothur and were then removed. Sequences were assigned to OTU using the cluster command and the average neighbour algorithm. All subsequent OTU-based analyses were performed using a cutoff of 0.03. Taxonomy was assigned to all remaining aligned sequences by comparing processed data to the silva128 databases for bacteria and archaea independently (arb -silva .de/ silva -license-information^66^).

### Histology

Following formalin fixation ileal tissues were dehydrated with increasing concentrations of ethyl-alcohol (50–100%) using an automated processer (Tissue-TEK VIP, Sakura, Finetek) and embedded in paraffin wax. Sections of 4 µm were cut using a microtome (Leitz RM2255), mounted on glass slides and stained with Hematoxylin and Eosin (H&E) using a Leica auto stainer (Leica ST5020) for histology examination using a light microscope (Leica DM3000).

### Immunohistochemistry (IHC)

For identification of the immune cells, formalin fixed paraffin embedded tissue sections of 4 µm mounted on to negatively charged glass slides were investigated by immunohistochemistry (IHC) using the manual technique with an ImmPRESS HRP anti-goat IgG (Iba1), anti-rabbit IgG (CD3) or anti-mouse-IgG (Pax5) (peroxidase) polymer detection kit (Vector Laboratories, Peterborough, UK). Primary antibodies specific for the macrophage marker Iba1 (AIFI/Iba1 goat anti-human polyclonal (internal) (unconjugated) antibody (Lifespan Biosciences, Nottingham, UK), no antigen retrieval, dilution 1 in 500), the T-cell marker CD3 (CD3 polyclonal rabbit anti-human antibody (Agilent Technologies Ltd, Cork, Ireland); antigen retrieval by citrate buffer at pH 6 heat treatment for 20 min (dilution 1 in 300) and the B-cell marker Pax5 (BD BioSciences, Oxford, England, monoclonal mouse antibody, antigen retrieval by citrate buffer at pH 6 heat treatment for 20 min (dilution 1 in 40) were used.

All sections, with or without antigen retrieval, were rinsed with H_2_O, and incubated with 3% (v/v) hydrogen peroxide, an endogenous enzyme block, for 30 min. Sections were rinsed with PBS before incubation in horse serum (from kit) for 30 min followed by incubation with the primary antibody. Sections were again rinsed with PBS, incubated with ImmPRESS goat, rabbit or mouse serum for 30 min and then rinsed again before application of the substrate chromogen, 3,3′-Diaminobenzidine (DAB). The DAB was washed off after approximately 40 s and the sections were counterstained with haematoxylin, rinsed and dehydrated before cover slipped.

### Villi measurements and cell counts

Measurements of villi length and width were taken on H&E stained ileal sections at 40× using a 1 cm graticule, with 10 well-orientated villi measured per section for length and width and 10 fields of vision assessed for frequency. Villus height was measured from the tip of the villus to the crypt-villus junction. Villus width was measured at the midpoint of the villus length, from the outside epithelial edges. Eosinophils, identified by their lobed nuclei and bright red large intracytoplasmic granules, and macrophages, and lymphocytes, identified by IHC, were examined at 400× magnification in the lamina propria mucosa. The cells of each cell type were counted in five representative full length villi in each case. Equally sized villi were chosen in all cases to allow comparison between the different age groups. In addition, T cells were counted in the mucosal epithelial lining. The composition of cells in the dome area was assessed separately.

### RNA extraction and cDNA synthesis

The frozen tissue samples of ileum were homogenised in TRIzol (Life Technologies, Paisley, UK) using a Pro Scientific homogeniser model PRO200 (PRO Scientific Inc., Oxford, CT, USA) with 3 s pulses repeated 10 times. RNA was then extracted and cleaned using the RNeasy Plus mini kit (Qiagen Ltd., Manchester, UK).

RNA quality and quantity were determined by measuring the absorbance at 260 nm using a NanoDrop spectrophotometer ND-100 (Thermo Scientific, Waltham, MA, United States), all samples had an absorbance above 1.8. RNA quality was further assessed on an Agilent Bioanalyser 2100 (Agilent Technologies Ireland Ltd., Dublin, Ireland) using the RNA 6000 Nano lab chip kit, all samples had RIN values between 8 and 10. cDNA was synthesised using a High Capacity cDNA Reverse Transcription kit (Applied Biosystems, Foster City, CA, USA) according to the manufacturer’s instructions. 2 µg of total RNA was reverse transcribed into cDNA using MultiScribe reverse transcriptase. The quantity of converted cDNA was determined by absorbance at 260 nm using a NanoDrop spectrophotometer and stored at – 20 °C.

### Quantitative PCR

Genes involved in the immune response, specifically genes for cytokines involved in recruiting and promoting immune cells were targeted and are listed in Supplementary Table 5. All primers for quantitative PCR (qPCR) were designed using Primer3 and analysed through BLAST to check specificity and homology to the bovine sequence. Primers for reference and target genes were commercially synthesised (Sigma-Aldrich Ireland, Dublin, Ireland). Five genes were assessed in the ileal tissue, all of which promoted cytokine expression in T cells or macrophages. The gene expression levels were measured by qPCR and the expression stabilities were evaluated by the M value calculated in GeNORM^67^.

Real-time PCR was carried out using an ABI 7500 Fast Real-time PCR System with SYBR green master mix (Applied Biosystems, Warrington, UK). Reactions were carried out in PCR tubes and prepared in a total volume of 20 µl with 2 µl cDNA, 10 µl SYBR green master mix, 7 µl nuclease free H_2_O and 1 µl of forward and reverse primer mix. Thermal cycling conditions remained constant for each assay, with an initial denaturation step at 95 °C for 15 min, followed by 40 cycles of denaturation at 95 °C for 5 s and annealing and extension at 60 °C for 40 s. SYBR green fluorescence was detected at the end of each cycle to track the quantity of PCR product. The efficiency of the qPCR reaction was calculated for each gene by creating a standard curve from two-fold serial dilutions of cDNA. For each gene, efficiency correction of the raw Ct values, normalisation to the reference genes, calculations of quantities relative to the max Ct value and log transformation of Ct values were all carried out using GenEx 5.2.1.3 (MultiD Analyses AB, Gothenburg, Sweden).

### Statistical analysis

Analysis of variance (ANOVA) with a Tukey HSD post-hoc test were carried out within the R software environment using the ‘lsmeans’ package to analyse differences between means for villi length, width, frequency and immune cell populations in the bovine ileum from birth to 96 days of life. Immune cell (eosinophil, macrophage and T cell) counts in the lamina propria of ileum harvested from calves were averaged and compared between age groups. Significant differences in gene expression levels in the ileum of calves were detected using one way ANOVA performed in Graphpad Prism (v7.02). For bacterial populations and immune cell changes across time, a 2 way ANOVA was performed using the same software.

Bacterial phylum, family and genus richness, evenness and diversity (Shannon–Wiener index and Simpson index) were calculated for each age cohort. ANOVA and post hoc, Tukey HSD tests were carried out within the R software environment using the ‘lsmeans’ package to analyze differences between means. Multivariate analysis was carried out using PRIMER-E v.7 software with the Distance-based permutational multivariate analysis of variance (PERMANOVA) add on^68^. Similarity matrices were constructed for samples using Bray–Curtis similarities on standardized, fourth root transformed abundance data. PERMANOVA was then performed to test the null hypothesis that there were no differences in microbial community structure across treatments at a significance level of α = 0.05 based on 9999 possible permutations. Nonmetric multidimensional scaling (nMDS) plots were constructed to visualize the data. Similarity percentages (SIMPER) were calculated using Bray–Curtis similarities to evaluate the level to which each phylum, family and genus contributed to the difference in community structures between groups. Effects and differences were declared as significant when P ≤ 0.05.

Pearson’s correlation coefficient (r) analysis was conducted to determine if any significant correlations could be observed between immune cell population levels in the villi and intra-epithelial layers in the ileum of calves at different age points and any of the most relatively abundant bacterial phyla and genera contributing to differences in bacterial community structure of calves between age points. This method was also used to determine if any statistically significant correlations existed between immune cell population profiles in the ileum villi of calves and gene expression levels of a subset of cytokines at different age points.

## Supplementary information

Supplementary Information.

## Data Availability

Sequence files associated with each sample have been submitted to the NCBI Sequence Read Archive (Accession number PRJNA453736).
