# Supplementary material for: Integrated analyses of the microbiological, immunological and ontological transitions in the calf ileum during early life
Source: Sci Rep. 2020 Dec 4;10:21264. doi: 10.1038/s41598-020-77907-0 (PMC7718239; doi:10.1038/s41598-020-77907-0)
Supplement: Supplementary file 1 — Supplementary Information. [file 41598_2020_77907_MOESM1_ESM.docx]

**Integrated analyses of the microbiological, immunological and ontological transitions in the calf ileum during early life**

Tamsin Lyons^1^, Hanne Jahns^2¶^, Joseph Brady^2^, Eóin O’Hara^3, 4^, Sinéad M. Waters^3^, David Kenny^3^, Evelyn Doyle^1^ and Kieran G. Meade^3,5¶*^

^1^ _Environmental Microbiology Group, School of Biology and Environmental Science & Earth Institute, University College Dublin, Belfield, Ireland_

^2^ _Pathobiology Section, School of Veterinary Medicine, University College Dublin, Dublin, Ireland_

^3^ _Animal & Bioscience Research Department, Animal and Grassland Research and Innovation Centre, Teagasc, Grange, County Meath, Ireland_

^4^ _Department of Agriculture, Food, and Nutritional Sciences, University of Alberta, Edmonton, Alberta, Canada._

^5^ _School of Agriculture and Food Science, University College Dublin, Dublin 4, Ireland._

**Supplementary Figures**


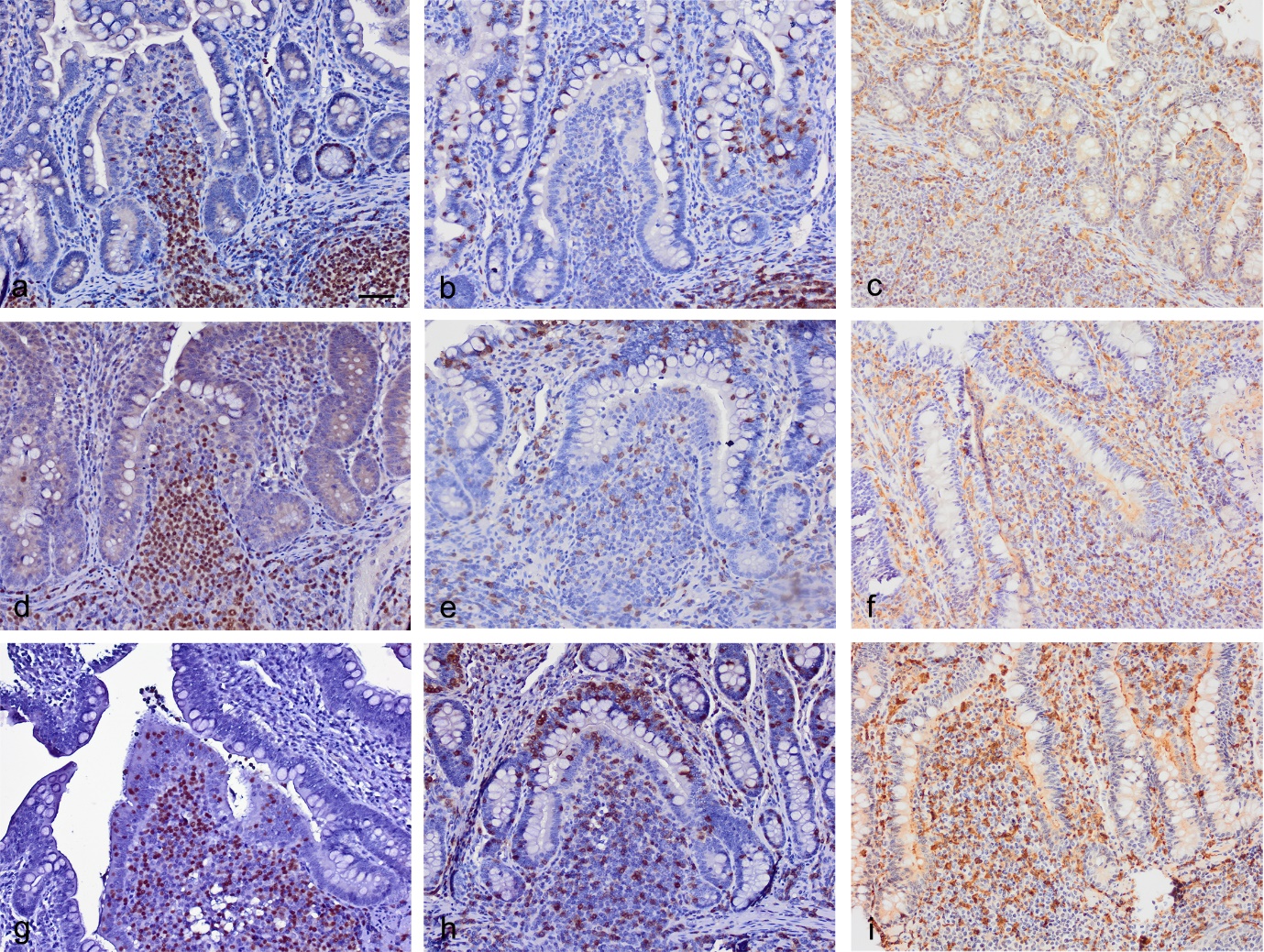


**Supplementary figure 1**: Calf dome area plate*;* Representative images of the ileal dome area of calves from 3 different age groups; D0 (a to c), D21 (d to f), D96 (g to i). Moderate numbers of B-cells are present in the lymphoid tissue of the dome area and infiltrating the FAE with a mild increase observed with age. First column, Anti-Pax5 immunohistochemical staining (brown), counterstained with Mayer’s haematoxylin. Single T-cells are visible in the dome area at D0 with an increase to moderate numbers with age. Second column, Anti-CD3 immunohistochemical staining (brown), counterstained with Mayer’s haematoxylin. Similarly, an increase of macrophages infiltrating the dome area is observed with age. Third column, Anti-Iba1 immunohistochemical staining (brown), counterstained with Mayer’s haematoxylin. Scale bar = 50 µm.


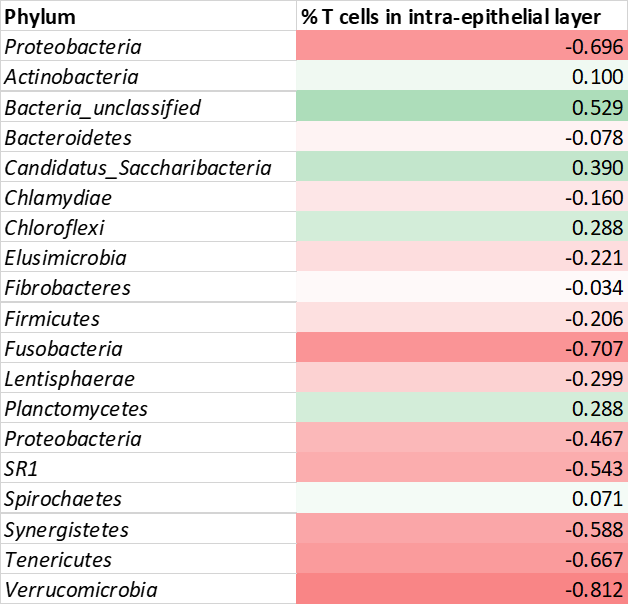


**Supplementary figure 2:** Heat map showing correlations between the proportion of T cells relative to total cells detected in the intra-epithelial layers of villi in the bovine ileum and the relative abundance of bacterial phyla in the ileum over the first 96 days of life and the. Pearson’s correlation coefficients (r) are given with r < 0 indicating a negative correlation (red), r = 0 indicating no correlation (white) and r > 1 indicating a positive correlation (green).

**Supplementary figure 3:** Heat map showing correlations between the relative abundance of sequences assigned to bacterial genera in the ileum and the percentage immune cells (macrophages, T cells & eosinophils) present in the ileal villi of calves over the first 96 days of life. Pearson’s correlation coefficients (r) are given with r < 0 indicating a negative correlation (red), r = 0 indicating no correlation (white) and r > 1 indicating a positive correlation (green).

**Supplementary figure 4:** Heat map showing correlations between the proportion of T cells relative to total cells detected in the intra-epithelial layers of villi in the bovine ileum and the relative abundance of bacterial genera in the ileum over the first 96 days of life and the Pearson’s correlation coefficients (r) are given with r < 0 indicating a negative correlation (red), r = 0 indicating no correlation (white) and r > 1 indicating a positive correlation (green).

**Supplementary figure 5A:** Heat map showing correlations between the abundance (%) of immune cells (macrophages, T cells & eosinophils) present in the ileal villi of calves over the first 96 days of life and the expression levels of genes associated with immune function. Pearson’s correlation coefficients (r) are given with r < 0 indicating a negative correlation (red), r = 0 indicating no correlation (white) and r > 1 indicating a positive correlation (green).

**Supplementary figure 5B:** Heat map showing correlations between the proportion of T cells relative to total cells detected in the intra-epithelial layers of villi in the bovine ileum and relative expression levels of genes associated with immune function in the ileum over the first 96 days of life and the Pearson’s correlation coefficients (r) are given with r < 0 indicating a negative correlation (red), r = 0 indicating no correlation (white) and r > 1 indicating a positive correlation (green).

**Supplementary tables**

**Supplementary table 1:** Mean relative abundance (%) of sequences assigned to bacterial phyla present in the ileum of calves at 6 sampling time points (Days 0, 7, 14, 21, 28 and 96) between birth and post-weaning. Unless shown all standard deviations were <0.001.

| **Phylum** | **Mean relative abundance (%)** | | | | | |
| --- | --- | --- | --- | --- | --- | --- |
|  | **D0** | **D7** | **D14** | **D21** | **D28** | **D96** |
| *Firmicutes* | 82.92(±5.22) | 53.24(±7.5) | 62.57(±4.26) | 52.83(±3.88) | 62.61(±6.14) | 86.25(±29.88) |
| *Proteobacteria* | 7.86(±0.5) | 11.25(±1.98) | 2.97(±0.33) | 7.89(±1.23) | 1.23(±0.1) | 1.64(±0.64) |
| *Verrucomicrobia* | 5.61 (±0.54) | 0.13(±0.02) | 0.89(±0.24) | <0.1 | <0.1 | <0.1 |
| *Bacteroidetes* | 1.96(±0.17) | 31.98(±3.7) | 17.52(±1.2) | 23.72(±3.71) | 6.35(±0.68) | 0.42(±0.12) |
| *Actinobacteria* | 0.81(±0.07) | 3.37(±0.57) | 15.78(±1.35) | 14.47(±1.28) | 28.63(±3.01) | 10.43(±1.89) |
| *Chlamydiae* | 0.39(±0.03) | <0.1 | <0.1 | 0.01 | <0.1 | 0.37(±0.15) |
| *Synergistetes* | 0.28(±0.03) | <0.1 | <0.1 | <0.1 | <0.1 | <0.1 |
| *Bacteria_*unclassified | <0.1 | <0.1 | <0.1 | <0.1 | <0.1 | <0.1 |
| *Fusobacteria* | <0.1 | <0.1 | <0.1 | <0.1 | <0.1 | <0.1 |
| *Fibrobacteres* | <0.1 | <0.1 | <0.1 | <0.1 | <0.1 | <0.1 |
| *Elusimicrobia* | <0.1 | <0.1 | <0.1 | <0.1 | <0.1 | <0.1 |
| *Spirochaetes* | <0.1 | <0.1 | <0.1 | <0.1 | <0.1 | <0.1 |
| *Tenericutes* | <0.1 | <0.1 | <0.1 | <0.1 | <0.1 | <0.1 |
| *Candidatus_*  *Saccharibacteria* | <0.1 | <0.1 | <0.1 | <0.1 | <0.1 | <0.1 |
| *Chloroflexi* | <0.1 | <0.1 | <0.1 | <0.1 | <0.1 | <0.1 |
| *SR1* | <0.1 | <0.1 | <0.1 | <0.1 | <0.1 | <0.1 |
| *Planctomycetes* | <0.1 | <0.1 | <0.1 | <0.1 | <0.1 | <0.1 |
| *Lentisphaerae* | <0.1 | <0.1 | 0.024 | <0.1 | <0.1 | <0.1 |
| *Armatimonadetes* | <0.1 | <0.1 | <0.1 | <0.1 | <0.1 | <0.1 |
| *Acidobacteria* | <0.1 | <0.1 | <0.1 | <0.1 | <0.1 | <0.1 |
| *Deinococcus-Thermus* | <0.1 | <0.1 | <0.1 | <0.1 | <0.1 | <0.1 |

**Supplementary table 2:** Mean relative abundance and similarity percentage (SIMPER) analysis of bacterial phyla accounting for approximately 70% of dissimilarity in bacterial community structures in the ileum of **A)** new born calves at day 0 (D0) versus calves at day 7 (D7); **B)** calves at day 7 (D7) versus day 14 (D14); **C)** calves at day 14 (D14) versus day 21 (D21); **D)** calves at day 21(D21) versus day 28 (D28); **E)** calves at day 28 (D28) versus day 96 (D96)

**A) Day 0 vs Day 7**

| **Phylum** | **Mean relative abundance (%)**  **D0** | **Mean relative abundance (%)**  **D7** | **Contribution to dissimilarity (%)** | **Cumulative % dissimilarity** |
| --- | --- | --- | --- | --- |
| ***Bacteroidetes*** | 1.13 | 2.11 | 19.7 | 19.7 |
| ***Verrucomicrobia*** | 1.44 | 0.59 | 13.82 | 33.52 |
| ***Chlamydiae*** | 0.73 | 0.07 | 10.81 | 44.33 |
| ***Synergistetes*** | 0.65 | 0.08 | 9.27 | 53.60 |
| ***Actinobacteria*** | 0.92 | 1.22 | 7.84 | 61.44 |
| ***Firmicutes*** | 3.01 | 2.66 | 5.64 | 67.07 |
| ***Fibrobacteres*** | 0.35 | 0.00 | 5.62 | 72.70 |
|  |  |  |  |  |

**B) Day 7 vs Day 14**

| **Phylum** | **Mean relative abundance (%)**  **D7** | **Mean relative abundance (%) D14** | **Contribution to dissimilarity (%)** | **Cumulative % dissimilarity** |
| --- | --- | --- | --- | --- |
| ***Actinobacteria*** | 1.22 | 1.99 | 17.85 | 17.85 |
| ***Bacteroidetes*** | 2.11 | 2.05 | 15.05 | 32.90 |
| ***Verrucomicrobia*** | 0.59 | 0.57 | 11.82 | 44.73 |
| ***Proteobacteria*** | 1.76 | 1.29 | 11.22 | 55.95 |
| ***Synergistetes*** | 0.08 | 0.36 | 7.17 | 63.12 |
| ***Fusobacteria*** | 0.13 | 0.38 | 5.65 | 68.78 |
| ***Firmicutes*** | 2.66 | 2.81 | 4.78 | 73.56 |
|  |  |  |  |  |

**C) Day 14 vs Day 21**

| **Phylum** | **Mean relative abundance (%) D14** | **Mean relative abundance (%) D21** | **Contribution to dissimilarity (%)** | **Cumulative % dissimilarity** |
| --- | --- | --- | --- | --- |
| ***Verrucomicrobia*** | 0.57 | 0.16 | 12.27 | 12.27 |
| ***Bacteroidetes*** | 2.05 | 2.03 | 11.48 | 23.75 |
| ***Bacteria_*unclassified** | 0.51 | 0.51 | 10.54 | 34.30 |
| ***Proteobacteria*** | 1.29 | 0.55 | 10.20 | 44.49 |
| ***Synergistetes*** | 0.36 | 0.06 | 7.95 | 52.45 |
| ***Fusobacteria*** | 0.38 | 0.09 | 6.80 | 59.24 |
| ***Spirochaetes*** | 0.11 | 0.32 | 5.20 | 64.44 |
| ***Elusimicrobia*** | 0.22 | 0.00 | 4.86 | 69.29 |
| ***Actinobacteria*** | 1.99 | 1.93 | 4.61 | 73.90 |
|  |  |  |  |  |

**D) Day 21 vs Day 28**

| **Phylum** | **Mean relative abundance (%) D21** | **Mean relative abundance (%) D28** | **Contribution to dissimilarity (%)** | **Cumulative % dissimilarity** |
| --- | --- | --- | --- | --- |
| ***Bacteroidetes*** | 2.03 | 1.46 | 17.33 | 17.33 |
| ***Proteobacteria*** | 1.55 | 0.96 | 14.57 | 31.90 |
| ***Actinobacteria*** | 1.93 | 2.25 | 10.02 | 41.92 |
| ***Fibrobacteres*** | 0.03 | 0.38 | 7.43 | 49.35 |
| ***Verrucomicrobia*** | 0.16 | 0.28 | 6.19 | 55.54 |
| ***Spirochaetes*** | 0.32 | 0.27 | 6.02 | 61.56 |
| ***Bacteria_*unclassified** | 0.96 | 0.89 | 5.20 | 66.76 |
| ***Chlamydiae*** | 0.17 | 0.27 | 5.14 | 71.89 |
|  |  |  |  |  |

**E) Day 28 vs Day 96**

| **Phylum** | **Mean relative abundance (%) D28** | **Mean relative abundance (%) D96** | **Contribution to dissimilarity (%)** | **Cumulative % dissimilarity** |
| --- | --- | --- | --- | --- |
| ***Bacteroidetes*** | 1.46 | 0.84 | 13.68 | 13.68 |
| ***Candidatus_Saccharibacteria*** | 0.12 | 0.63 | 11.50 | 25.19 |
| ***Actinobacteria*** | 2.25 | 1.89 | 10.84 | 36.02 |
| ***Chlamydiae*** | 0.27 | 0.66 | 8.77 | 44.80 |
| ***Fibrobacteres*** | 0.38 | 0.24 | 6.10 | 50.89 |
| ***Proteobacteria*** | 0.96 | 1.07 | 6.05 | 56.94 |
| ***Spirochaetes*** | 0.27 | 0.35 | 5.88 | 62.82 |
| ***Elusimicrobia*** | 0.17 | 0.34 | 5.27 | 68.09 |
| ***Firmicutes*** | 2.78 | 2.99 | 4.47 | 72.56 |

**Supplementary table 3:** Bacterial richness, evenness and diversity measurements in the ileum of calves at 6 time points (Days 0, 7, 14, 21, 28 and 96) between birth and post-weaning.

| **Group** | **Species richness** | **Species evenness** | **Shannon-Wiener index** |
| --- | --- | --- | --- |
| **D0** | 40.02 ± 3.95^ab^ | 0.94 ± 0.005^ab^ | 4.83 ± 0.11^ab^ |
| **D7** | 25.56 ± 0.84^c^ | 0.93 ± 0.003^c^ | 4.27 ± 0.04^c^ |
| **D14** | 45.56 ± 5.41^a^ | 0.93 ± 0.003^c^ | 4.91 ± 0.11^a^ |
| **D21** | 30.93 ± 7.6^bc^ | 0.94 ± 0.007^bc^ | 4.56 ± 0.26^bc^ |
| **D28** | 40.7 ± 10.8^ab^ | 0.94 ± 0.008^ab^ | 4.84 ± 0.31^ab^ |
| **D96** | 34.91 ± 6.05^abc^ | 0.94 ± 0.009^abc^ | 4.65 ± 0.11^abc^ |

**Supplementary table 4:** Cell density (counted as total number of cells per field of view) in the rumen, jejunum, ileum and colon of new born calves (D0) and in calves throughout early life at days 7 (D7), 14 (D14), 21 (D21), 28 (D28) and 96 (D96) after birth. In total 18 calves were evaluated, 3 per age group, with 10 fields of view assessed for each tissue sample. ^a-d^Means within a column that do not share a common superscript letter are significantly different (p  <  0.05), as analysed by one-way ANOVA.

| **Day** | **Rumen** | **Jejunum** | **Ileum** | **Colon** |
| --- | --- | --- | --- | --- |
| D0 | 32.4 ± 1.7^a^ | 28.5 ± 1.4^a^ | 29.7 ± 3^a^ | 29.7 ± 2^a^ |
| D7 | 38.9 ± 3.9^b^ | 29.8 ± 1.5^ab^ | 31.3 ± 2.3^a^ | 30.1 ± 2^a^ |
| D14 | 33 ± 2.3^a^ | 29.9 ± 1.8^ab^ | 34.1 ± 2.6^b^ | 30.6 ± 1.9^a^ |
| D21 | 29.6 ± 1.4^cd^ | 30.5 ± 2^b^ | 38.5 ± 2.1^c^ | 29.8 ± 1.9a |
| D28 | 30.5 ± 1.6^d^ | 30.6 ± 1.7^b^ | 39.4 ± 3.5^c^ | 29.4 ± 1.8^a^ |
| D96 | 28.6 ± 2.1^c^ | 37.2 ± 3.3^c^ | 43.5 ± 2d | 30.3 ± 1.6^a^ |

**Supplementary table 5:** Oligonucleotide qPCR primers (checked for efficacy and successfully used to target the listed host genes in other bovine tissues) that target selected host genes (IL-33, IL-1α, IL-1β, TGF-β, IL-10, IL-8 and NLRP3) associated with immune system responses and genes with roles in basic cell metabolism (RPS15, GAPDH, SDHA and GUSB) for use as reference genes.

| Gene name | Forward primer | Reverse primer |
| --- | --- | --- |
| *IL1α* | AGCCAGTGGGAAGATTCTGA | GCATTCCTGGTGGATBACTC |
| *Il1ß* | CCCTGCAGCTGGAGGAAGTA | CTTCGATTTGAGAAGTGCTGATGT |
| *Il8* | CATTCCACACCTTTCCACCC | CCTTCTGCACCCACTTTTCC |
| *IL10* | AGAACCACGGGCCTGACA | ACCGCCTTGCTCTTGTTTTC |
| *IL33* | ACAGATGATGGTGGCAGTCA | AAACACACTGGGAGGACGTC |
| *TGFb* | ACCCTCGGAAAATGCCATCC | CTGAACTCGGCCTTCACCAA |
| *NLRP3* | CCTTCTGGACTCTGACCGGG | TCTGTCTGACCCCGAGGAAT |
| *RPS15* | GCGACATGATCATTCTACCCG | GGTAGTGGCCGATCATCTCA |
| *GAPDH* | CTCCCAACGTGTCTGTTGTG | TGAGCTTGACAAAGTGGTCG |
| *SDHA* | AAGACGTTCGACAGGGGAAT | ACTCGTCAACCCTCTCCTTG |
| *GUSB* | ACCATCGCCATCAACAACAC | TCCCGCGTAGTTGAAGAAGT |
